# Acute pulmonary embolism and cancer: findings from the COPE study

**DOI:** 10.1007/s00392-023-02323-z

**Published:** 2023-11-15

**Authors:** Cecilia Becattini, Ludovica Anna Cimini, Giorgio Bassanelli, Aldo P. Maggioni, Fulvio Pomero, Ilaria Lobascio, Iolanda Enea, Daniela P. Pomata, Maria Pia Ruggieri, Beniamino Zalunardo, Anna Novelli, Stefania Angela Di Fusco, Marco Triggiani, Marco Marzolo, Chiara Fioravanti, Giancarlo Agnelli, Lucio Gonzini, Michele M. Gulizia, on behalf of COPE Investigators

**Affiliations:** 1 Internal, Vascular and Emergency Medicine-Stroke Unit, University of Perugia, Piazzale Lucio Severi 1, 06129 Perugia, Italy; 2 Department of Cardiology, Ospedale Alessandro Manzoni, Lecco, Italy; 3 ANMCO Research Center, Heart Care Foundation, Florence, Italy; 4 Department of Internal Medicine, Ospedale Michele e Pietro Ferrero, Verduno, Italy; 5 Department of Cardiology, Ospedale Civile, Arzignano, Italy; 6 U.O.C. Medicina e Chirurgia d’Urgenza, A.O.R.N. “S. Anna e S. Sebastiano”, Caserta, Italy; 7 Medicina d’Urgenza e Pronto Soccorso, Ospedale Policlinico S. Orsola-Malpighi, Bologna, Italy; 8 U.O.C. Medicina d’Urgenza e Pronto Soccorso, AO San Giovanni Addolorata, Rome, Italy; 9 Angiology Unit, Azienda ULSS 2 Marca Trevigiana, Castelfranco Veneto, Treviso, Italy; 10 Pronto Soccorso e Medicina d’Urgenza, Ospedali Riuniti, Livorno, Italy; 11 Cardiologia Clinica e Riabilitativa, P.O. San Filippo Neri, Rome, Italy; 12 U.O. Cardiologia, Ospedale Civile “La Memoria”, Gavardo, Brescia Italy; 13 U.O.C. Medicina Interna-Angiologia, Ospedale S. Maria Della Misericordia, Rovigo, Italy; 14 Medicina Interna, Ospedali Galliera, Genoa, Italy; 15 Division of Cardiology, Garibaldi-Nesima Hospital, Catania, Italy

**Keywords:** Pulmonary embolism, Cancer-associated thromboembolism, Cancer, Anticoagulant, DOAC

## Abstract

**Background:**

Patients with acute venous thromboembolism associated with cancer have an increased risk of recurrences and bleeding in the long term.

**Research question:**

To describe the clinical features and short-term course of patients with acute pulmonary embolism (PE) and active cancer, previous cancer or no cancer.

**Study design and methods:**

Patients with acute PE included in COPE—prospective, multicentre study of adult patients with acute, symptomatic, objectively diagnosed PE—were classified as having active cancer, previous cancer, or no cancer.

**Results:**

Overall, 832 patients had active cancer, 464 with previous cancer and 3660 patients had no cancer at the time of acute PE. The most prevalent primary sites of active cancer were urogenital (23.0%), gastrointestinal (21.0%), and lung (19.8%), with a high prevalence of metastatic disease (57.6%) and ongoing anticancer treatment (16.2%). At discharge, a direct oral anticoagulant was used in 43.1%, 78.8%, and 82.0% of patients with active cancer, previous cancer, and no cancer, respectively. Rates of death in-hospital and at 30 days were higher in patients with active cancer compared to patients with previous cancer and no cancer (7.9% vs. 4.3% vs. 2.2% and 13.8% vs. 5.2% vs. 2.6%, respectively). Rates of major bleeding were 4.8%, 2.6%, and 2.4%, respectively. Among patients with active cancer, lung or metastatic cancer were independent predictors of death; brain, hematological or gastrointestinal cancer had the highest risk of major bleeding.

**Interpretation:**

Among patients with acute PE, those with active cancer have high risks for death or major bleeding within 30 days. These risks vary based on primary site of cancer.

*Clinical trial registration*: clinicaltrial.gov identifier: NCT03631810.

**Graphical abstract:**

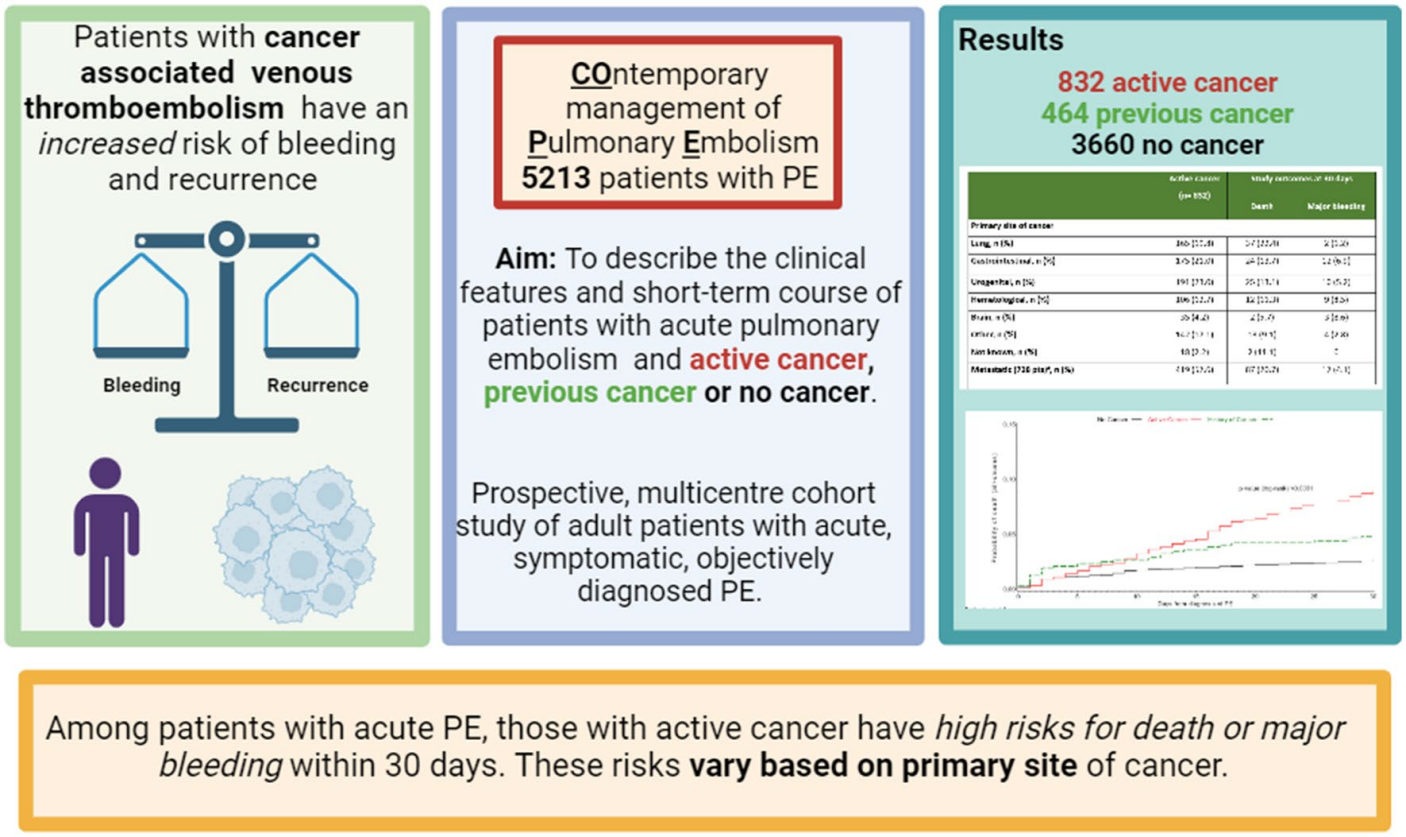

**Supplementary Information:**

The online version contains supplementary material available at 10.1007/s00392-023-02323-z.

## Introduction

Venous thromboembolism (VTE), presenting either as deep vein thrombosis or pulmonary embolism (PE), is a common event in patients with cancer [1, 2]. In fact, several connections have been described between cancer and a procoagulant status at a biochemical level [3]. In addition, invasive procedures, anticancer treatments, as well as hospital stay or reduced mobility frequently occur in the life-course of cancer patients and are all associated with an increased risk of VTE [4]. In comparison to non-cancer patients, patients with cancer and VTE have an increased risk of recurrent VTE and major bleeding during anticoagulant treatment [5, 6]. In patients with cancer, a diagnosis of VTE also seems to have an impact in survival at 3 months [7]. For these reasons, the management of VTE in cancer patients is challenging and dedicated clinical studies have been conducted in this specific setting. According to the results of these studies, the management of VTE in cancer patients has changed during the last decades.

Current international guidelines for the treatment of VTE identify direct oral anticoagulants (DOACs) as the treatment of choice for the majority of patients with cancer [8–12]. A special caution and eventually treatment with low molecular weight heparins (LMWH) is recommended for patients with high risk for bleeding. However, the majority of the available evidence deals with the long-term phase or excludes specific groups of cancer patients. As a result, only limited data on the contemporary initial management and course of the disease in cancer patients with acute VTE are available.

We recently performed the COPE study, a multicenter registry to describe contemporary management strategies and course of patients with acute PE [13]. The present analysis of patients included in the COPE study is aimed at describing the clinical features, contemporary management, and short-term course of patients with acute PE, by the presence of active cancer, previous cancer, or no cancer.

## Study design and methods

The COPE study (clinicaltrial.gov identifier: NCT03631810) is a prospective, multicentre cohort study of adult patients with acute, symptomatic, objectively diagnosed PE (either first or recurrent episode) [13]. The study was conducted in accordance with the Declaration of Helsinki and the protocol was approved by the Institutional Review Board at the coordinating center and then at each site according to local policies and procedures.

Patients were enrolled at Cardiology, Acute Care Medicine and Internal Medicine Departments in Italy. Diagnostic work-out, risk stratification, and treatment strategies were at the discretion and responsibility of the attending physicians.

The study was supported by an unrestricted grant from Daiichi Sankyo Europe and Daiichi Sankyo Italy.

Patients aged 18 years or older with symptomatic objectively confirmed acute PE were included after the release of informed consent from April 2018 to December 2020. Patients were excluded from the COPE study in case of participation in controlled trials on the management of acute PE.

For the purpose of the present analysis, patients were classified as having active cancer if they had a diagnosis of malignancy in the 6 months before index PE or at study inclusion, metastatic disease or were receiving anticancer treatment. Patients with data unavailable on presence/absence of cancer were excluded from this analysis.

Patients were evaluated at the time of diagnosis, at discharge, and at 30 days (± 4) from the index PE. For patients discharged beyond 30 days from index PE, the study end was considered at 30 days.

### Study outcomes

The co-primary outcomes of the study were in-hospital death and death at 30 days [13]. The cause of death was reported by the investigators and adjudicated by a central and independent Clinical Event Committee unaware of physician classification. PE-related death was defined as one of the following: death occurring within 1 week from diagnosis where PE was the most probable cause or diagnosis based on objective diagnostic testing performed before death or as assessed at autopsy.

The primary safety outcome was major bleeding according to ISTH criteria, occurring up to 30 days from the index PE [14].

### Data collection

Data on demographic patient features, risk factors for VTE, clinical status, imaging, and laboratory results were electronically collected at presentation, at discharge, and at 30 days (± 4) after the index event via a secure website. For the index PE event, the following were collected: patient characteristics, medical history, predisposing risk factors, provoking risk factors (within the previous 4 weeks), symptoms, extent and location of PE, and date and method of diagnosis. During the hospital stay, at the discharge, and at 30 days, information about vital status, vital signs, anticoagulant treatment, and thromboembolic and bleeding events were collected.

### Statistical analysis

Categorical variables are presented as numbers and percentages and compared among the groups of patients with active cancer, previous cancer, or no cancer by Chi-square-test; continuous variables are presented as means and standard deviations (SD) and compared by analysis of variance (ANOVA) if normally distributed, or by Kruskal–Wallis test, if not.

Multivariable regression analyses (Cox model, backward selection) were performed to identify independent predictors of 30-day all-cause death in patients with and without active cancer. Multivariable analyses were constructed from the set of significant (*P* < 0.10) univariable predictors at entry and of covariates of clinical interest. The following variables were inserted in the Cox models: age (continuous), systolic blood pressure (continuous), and heart rate (continuous), gender, previous VTE, bed rest > 3 days < 4 weeks prior of EP (yes, no (reference group (RG)), unknown), hospitalization < 4 weeks prior of EP, chronic obstructive pulmonary disease, chronic heart failure, cognitive impairment, abnormal saturation (oxygen saturation < 90% in room air or < 95% with oxygen), respiratory rate [≥ 30, 29–20, < 20 (RG), unknown], reduced urinary output (yes, no (RG), not assessed), vigilance status abnormal due to PE. Gender was added to multivariable models as variable of clinical interest. Among patients with active cancer, an additional model was performed, considering site of cancer, metastatic cancer, and ongoing chemotherapy as additional covariates; results are reported as hazard ratios (HR) with 95% confidence interval (CI).

Finally, Kaplan–Meier estimates for death at 30 days from diagnosis of PE were calculated in the patients with active cancer, previous cancer, or no cancer and compared by log-rank test. Either estimates unadjusted or adjusted for PE severity according to ESC guidelines 2014 were calculated.

Analyses were performed with SAS system software, version 9.4.

## Results

Data on cancer were available in 4956 out of 5213 patients included in the COPE study (95.1%); of these patients, 832 (16.8%), 464 (9.4%), and 3660 (73.8%) were classified as having active cancer, previous cancer, or no cancer, respectively. The main features of study patients by presence of active cancer, previous cancer, or no cancer are reported in Table 1**.**

**Table 1:** Main characteristics of study patients by cancer status

Patient feature	Total population (*n* = 4956)	Active cancer (*n* = 832)	Previous cancer (*n* = 464)	No cancer (*n* = 3660)	*P*
Age (years), mean ± SD	70 ± 16	71 ± 12	76 ± 10	69 ± 17	< 0.0001
Range	18–100	19–100	38–97	18–99	
< 65, *n* (%)	1550 (31.3)	233 (28.0)	63 (13.6)	1254 (34.3)	< 0.0001
> 80, *n* (%)	1421 (28.7)	200 (24.0)	177 (38.2)	1044 (28.5)	< 0.0001
Male sex, *n* (%)	2373 (47.9)	439 (52.8)	211 (45.5)	1723 (47.1)	0.007
Risk factors for VTE					
Previous VTE, *n* (%)	836 (16.9)	110 (13.2)	86 (18.5)	640 (17.5)	0.007
Surgery < 4 weeks, *n* (%)	336 (6.8)	71 (8.5)	20 (4.3)	245 (6.7)	0.01
Trauma < 4 weeks, *n* (%)	368 (7.4)	19 (2.3)	22 (4.7)	327 (8.9)	< 0.0001
Major Trauma < 4 weeks, *n* (%)	89 (1.8)	2 (0.2)	4 (0.9)	83 (2.3)	0.0001
Bed rest > 3 days < 4 weeks, *n* (%)	1057 (21.3)	187 (22.5)	90 (19.4)	780 (21.3)	0.43
Hospitalization < 4 weeks, *n* (%)	691 (13.9)	143 (17.2)	61 (13.2)	487 (13.3)	0.01
Hormonal treatment, *n* (%) on 2583 females	198 (7.7)	9 (2.3)	5 (2.0)	184 (9.5)	< 0.0001
Pregnancy, *n* (%) on 2583 females	23 (0.9)	0 (0.0)	1 (0.4)	22 (1.1)	0.06
Comorbidities					
COPD, *n* (%)	605 (12.2)	100 (12.0)	68 (14.7)	437 (11.9)	0.24
CHF, *n* (%)	346 (7.0)	50 (6.0)	45 (9.7)	251 (6.9)	0.04
Hemodialysis, *n* (%)	15 (0.3)	1 (0.1)	1 (0.2)	13 (0.4)	0.51
Cirrhosis, *n* (%)	44 (0.9)	18 (2.2)	5 (1.1)	21 (0.6)	< 0.0001
Cognitive impairment, *n* (%)	491 (9.9)	64 (7.7)	43 (9.3)	384 (10.5)	0.045
Obesity (4806 pts)^a^, *n* (%)	1010 (21.0)	119 (14.8)	80 (18.0)	811 (22.8)	< 0.0001
Major bleeding < 4wks, *n* (%)	75 (1.5)	16 (1.9)	6 (1.3)	53 (1.5)	0.55
HIT (4842 pts)^a^, *n* (%)	9 (0.2)	3 (0.4)	0 (0.0)	6 (0.2)	0.31
COVID-19, *n* (%)	94 (1.9)	4 (0.5)	5 (1.1)	85 (2.3)	0.0005
Laboratory parameters at admission					
Hemoglobin, g/dl, mean ± SD	13.0 ± 2.0	11.9 ± 2.1	13.1 ± 1.8	13.2 ± 2.0	< 0.0001
≤ 10 g/dl, *n* (%)	439 (8.9)	156 (18.9)	29 (6.3)	254 (7.0)	< 0.0001
< 10 g/dl, *n* (%)	396 (8.0)	147 (17.8)	28 (6.1)	221 (6.1)	< 0.0001
Creatinine clearance, (4244 pts)^a^ ml/min, mean ± SD	71.1 ± 26.8	72.1 ± 26.3	63.6 ± 23.5	71.8 ± 27.2	< 0.0001
60–45 ml/min, *n* (%)	995 (23.4)	159 (21.8)	106 (26.1)	730 (23.5)	0.26
44–30 ml/min, *n* (%)	497 (11.7)	85 (11.7)	64 (15.8)	348 (11.2)	0.03
< 30 ml/min, *n* (%)	193 (4.6)	26 (3.6)	26 (6.4)	141 (4.5)	0.09
Management setting					
Emergency department	1537 (31.0)	295 (35.4)	153 (32.9)	1089 (29.7)	< 0.0001
Cardiology department	1395 (28.1)	165 (19.8)	122 (26.3)	1108 (30.3)	
Internal medicine	1129 (22.8)	206 (24.8)	108 (23.3)	815 (22.3)	
Other	895 (18.1)	166 (20.0)	81 (17.5)	648 (17.7)	
Contraindications for anticoagulation, *n* (%)	103 (2.1)	41 (4.9)	8 (1.7)	54 (1.5)	< 0.0001

VTE, Venous thromboembolism; COPD, Chronic obstructive pulmonary disease; CHF, congestive heart failure; HIT, heparin-induced thrombocytopenia

^a^Percentages were evaluated on patients with data available reported in brackets for each variable

Patients with previous cancer were older than patients with active cancer or no cancer, with a higher proportion of patients aged over 80 years. The prevalence of risk factors for PE differed by cancer status. Patients with active cancer had higher prevalence of recent surgery (surgery 8.5% vs. 4.3% vs. 6.7%) or recent hospitalization (17.2% vs. 13.2% vs. 13.3%) and lower prevalence of recent trauma (2.3% vs. 4.7% vs. 8.9%) and previous VTE (13.2% vs. 18.5% vs. 17.5%) compared to patients with previous cancer and to patients with no cancer. Similarly, a different distribution of comorbidities was observed among the three patient groups. Of note, patients with active cancer had about threefold higher prevalence of severe anemia at admission (17.8% vs. 6.1% vs. 6.1%) in comparison to other patient groups. Finally, active cancer was associated with higher prevalence of contraindications for anticoagulation.

The most prevalent primary sites of active cancer were urogenital (23.0%), gastrointestinal (21.0%), and lung (19.8%), with a high prevalence of patients with metastatic disease (57.6%) and on ongoing anticancer therapy (Table 2).

**Table 2:** Main features of active cancer

Site of active cancer (832 patients)	Active cancer *n* (%)
Lung, *n* (%)	165 (19.8)
Gastrointestinal, *n* (%)	175 (21.0)
Urogenital, *n* (%)	191 (23.0)
Hematological, *n* (%)	106 (12.7)
Brain, *n* (%)	35 (4.2)
Other, *n* (%)	142 (17.1)
Not known, *n* (%)	18 (2.2)
Metastatic (728 pts), *n* (%)	419 (57.6)
Ongoing chemotherapy (791 pts)^a^, *n* (%)	364 (46.0)
Ongoing radiotherapy (788 pts)^a^, *n* (%)	128 (16.2)

^a^Percentages were evaluated on patients with data available reported in brackets for each variable

The prevalence of PE symptoms in the groups of patients with active cancer, previous cancer, or no cancer is reported in Supplementary Table S1.

Among patients with active cancer, 268 (32.2%) had cancer as the only risk factor for PE, while 129 (15.5%) had four or more risk factors for PE. Previous cancer was the sole risk factor in 187 patients (40.3%) among those with previous cancer. Among patients without cancer, 1200 (32.8%) suffered PE in the absence of any identifiable risk factor and 189 (5.2%) had four or more risk factors for PE.

### Diagnosis and risk stratification

In almost all patients, the diagnosis of PE was obtained by computed tomography pulmonary angiogram (Supplementary Table S2). Patients with active cancer more frequently had the most proximal localization of PE at the segmental level and less commonly isolated subsegmental localization compared to patients with previous cancer and without cancer.

A lower proportion of patients with active cancer received lower limb ultrasonography or echocardiography in comparison to patients with previous cancer or without cancer.

A concomitant deep vein thrombosis was confirmed at lower limb ultrasonography in a larger proportion of patients with active cancer compared to the other patient groups (68.1% vs. 59.5% vs. 60.1%).

According to ESC guidelines, no patient with cancer was classified at low risk for death (Supplementary Table S3). However, the prevalence of intermediate–low-risk patients was about doubled among patients with active cancer in comparison to those with previous cancer or without cancer; the prevalence of high-risk patients was similar across different patient groups, while that of intermediate–high risk was higher in patients with previous cancer.

### Treatment of PE

During hospitalization, a lower proportion of patients with active cancer received oral anticoagulants in comparison to patients with previous cancer or no cancer (Table 3). At discharge, 43.1% of patients with active cancer, 78.8% of those with previous cancer and 82.0% of those without cancer received a DOAC for the treatment of PE.

**Table 3:** Use of anticoagulant agents during hospital stay and at discharge by cancer status

	Total population (*n* = 4956)	Active cancer (*n* = 832)	Previous cancer (*n* = 464)	No cancer (*n* = 3660)	*P*
Anticoagulant during hospital stay					
≥ 1 parenteral anticoagulant°, *n* (%)	4753 (92.3)	787 (94.6)	432 (93.1)	3354 (91.6)	0.01
DOACs, *n* (%)	2469 (49.8)	214 (25.7)	235 (50.7)	2020 (55.2)	< 0.0001
VKAs, *n* (%)	264 (5.3)	18 (2.2)	30 (6.5)	216 (5.9)	< 0.0001
Revascularization during hospital stay					
Thrombolysis, *n* (%)	267 (5.4)	28 (3.4)	17 (3.7)	222 (6.1)	0.002
* Percutaneous, n (%)*	*38 (14.2)*	*5 (17.9)*	*2 (11.8)*	*31 (14.0)*	*0.42*
* Systemic, n (%)*	*226 (84.6)*	*22 (78.6)*	*15 (88.2)*	*189 (85.1)*	*0.43*
Contraindication for thrombolysis, *n* (%)	418 (8.4)	156 (18.8)	42 (9.1)	220 (6.0)	< 0.0001
Vena cava filter, *n* (%)	50 (1.0)	17 (2.0)	3 (0.7)	30 (0.8)	0.004

	Total population (*n* = 4555)	Active cancer (*n* = 706)	History of cancer (*n* = 420)	No cancer (*n* = 3429)	*P*
Anticoagulants at discharge^a^					
Parenteral agents, *n* (%)	859 (18.9)	385 (54.5)	57 (13.6)	417 (12.2)	< 0.0001
DOACs, *n* (%)	3445 (75.6)	304 (43.1)	331 (78.8)	2810 (82.0)	< 0.0001
VKAs, *n* (%)	299 (6.6)	18 (2.6)	34 (8.1)	247 (7.2)	< 0.0001
Oral agents, *n* (%)	3744 (82.2)	322 (45.6)	365 (86.9)	3057 (89.2)	< 0.0001

Results in italics describe subgroups of patients receiving thrombolytic treatment by different routes of administration (percutaneous or systemic)

°Use of variable sequences of UFH, LMWH, and/or fondaparinux in every single patient was reported

^a^Among 4555 patients discharged alive from hospital within 30 from EP diagnosis

Thrombolytic treatment, either systemic or percutaneous, was administered to 3.4% of patients with active cancer, 3.7% with previous cancer, and to 6.1% of patients without cancer (Table 3). Among patients who received thrombolysis, percutaneous techniques were performed in 5 patients with active cancer (17.9%), in 2 patients with previous cancer (11.8%), and in 31 patients with no cancer (14.0%). Contraindications for thrombolysis were reported in 18.8%, 9.1%, and 6.0% of patients with active cancer, previous cancer, and without cancer, respectively.

The use of individual anticoagulant or thrombolytic strategies by primary site of cancer is reported in Supplementary Table S4.

### Outcomes by presence of cancer and by type of cancer

**Fig. 1:**
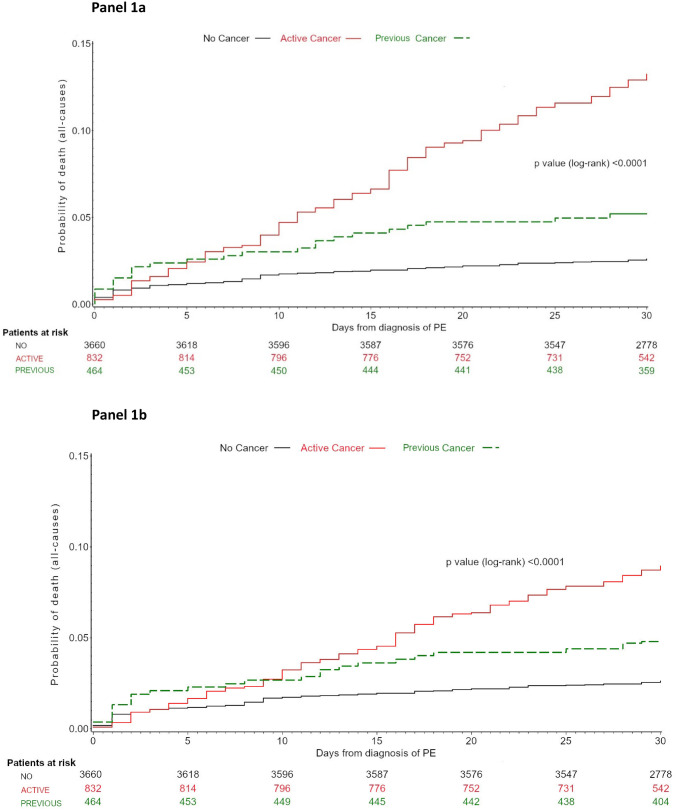
Kaplan–Meier curve for time to all-cause death at 30 days by cancer status, unadjusted (panel A) and after adjusting for PE severity according to ESC guidelines 2014 (panel B)

Both, in-hospital death and death at 30 days occurred in higher proportions of patients with active cancer in comparison to patients with previous cancer or without cancer (7.9% vs. 4.3% vs. 2.2%, in-hospital and 13.8% vs. 5.2% vs. 2.6% at 30 days, respectively) (Table 4; Fig. 1). The risk for death at 30 days was higher in patients with active cancer in comparison to patents without cancer (HR 5.51, 95% CI 4.20–7.23) and to those with previous cancer (HR 2.75, 95% CI 1.78–4.27).

**Table 4:** Clinical outcome by cancer status

	Total population (*n* = 4956)	Cancer status			*P*
		Active (*n* = 832)	Previous (*n* = 464)	No cancer (*n* = 3660)	
In-hospital death, *n* (%)	168 (3.4)	66 (7.9)	20 (4.3)	82 (2.2)	< 0.0001
Death at 30 days, *n* (%)	235 (4.7)	115 (13.8)	24 (5.2)	96 (2.6)	< 0.0001
In-hospital major bleeding, *n* (%)	131 (2.6)	35 (4.2)	10 (2.2)	86 (2.4)	0.008
Major bleeding at 30 days, *n* (%)	147 (3.0)	40 (4.8)	12 (2.6)	95 (2.4)	0.003

These data were confirmed after adjusting for the severity of PE (Fig. 1, panel B).

**Fig. 2:**
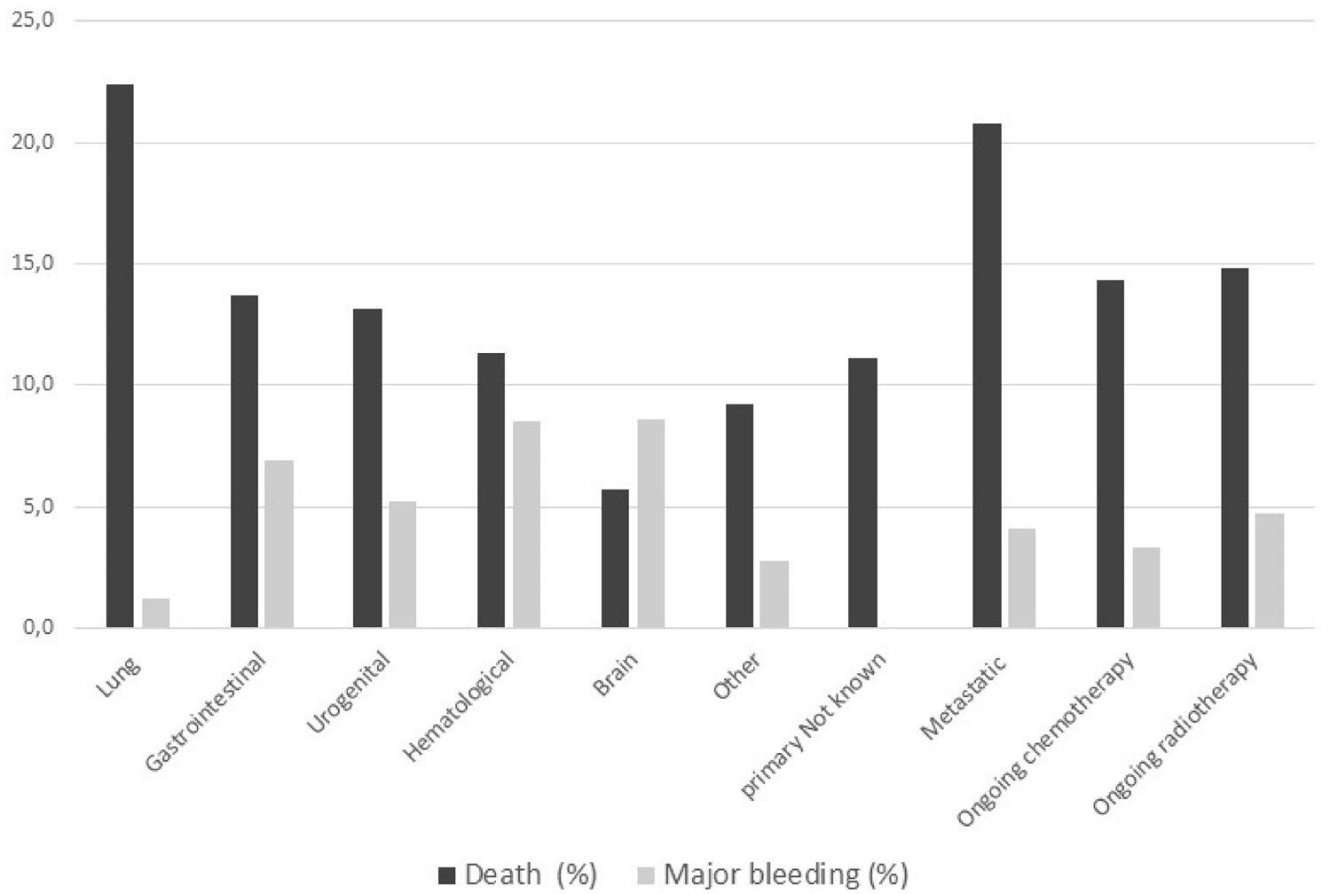
Clinical outcome at 30 days in patients with active cancer by site and stage of cancer

In patients with active cancer, the cause of death at 30 days was cancer itself in 66 (57.4%), PE in 19 (16.5%), and major bleeding in 6 (5.2%). These proportions were 1.7%, 52.5%, and 3.3% in patients without active cancer. In patients with active cancer, the rates and causes of death differed by primary site of cancer (Fig. 2; Supplementary Table S5). Patients with lung cancer had the highest mortality at 30 days (22.4%), and this was mainly due to cancer (67.6%); patients with hematological malignancy died due to acute PE or major bleeding in 25% each and none of these patients died due to cancer.

Major bleeding occurred in a higher proportion of patients with active cancer in comparison to those with previous cancer or without cancer, both during the in-hospital phase and at 30 days (4.2% vs. 2.2% vs. 2.4% in-hospital and 4.8% vs. 2.6% vs. 2.4% at 30 days, respectively). In patents with active cancer, only one major bleeding was an intracranial hemorrhage (ICH) that occurred in a patient with hematologic malignancy (Supplementary Table S6). Patients with hematological or brain cancer had the highest incidences of major bleeding. Of note, all major bleeding in patients with gastrointestinal cancer occurred at the gastrointestinal level, while no ICH was observed in patients with brain cancer.

Risk factors for death differed among patients with active cancer, previous cancer, or no cancer. Increased heart rate, reduced urinary output, advanced age, and history of heart failure were independent predictors for death, both in patients with and without active cancer (Table 5). In patients with active cancer, primary lung cancer and metastatic disease at the time of PE were additional independent predictors of death at 30 days.

**Table 5:** Predictors of death at 30 days in patients with or without active cancer as assessed by Cox proportional hazard models (backward selection)

	Patients with active cancer Model I (*n* = 832)	Patients with active cancer Model II (*n* = 832)	Patients without active cancer (*n* = 4124)
Variables	*P*	*P*	P
	HR (95% CI)	HR (95% CI)	HR (95% CI)
Age, years	0.018	0.004	< 0.0001
	1.02 (1.01–1.04)	1.03 (1.01–1.05)	1.04 (1.02–1.05)
Systolic blood pressure, mmHg	NS	–	0.0215
			0.991 (0.98–0.99)
Heart rate, beats per min	< 0.0001	< 0.0001	0.0115
	1.01 (1.01–1.02)	1.01 (1.01–1.02)	1.007 (1.00–1.01)
Respiratory rate^a^, breaths per min	NS	–	0.0207
20–29 versus < 20			0.97 (0.56–1.67)
≥ 30 versus < 20			2.18 (1.17–4.03)
Unknown versus < 20			1.42 (0.86–2.37)
Reduced urinary output^b^	0.0004	0.0102	0.0211
Yes versus no	2.98 (1.71–5.20)	2.39 (1.31–4.36)	2.05 (1.23–3.43)
Not assessed versus no	0.89 (0.47–1.68)	0.84 (0.44–1.62)	1.15 (0.65–2.04)
Abnormal vigilance status due to PE	NS	–	0.0028
Yes versus no			2.13 (1.30–3.49)
> 3 days in bed ≤ 4 weeks prior to PE^c^	NS	0.0331	0.0088
Yes versus no;		1.61 (1.06–2.43)	1.81 (1.21–2.71)
Unknown versus no		2.14 (0.94–4.87)	2.12 (0.95–4.72)
COPD	0.0043	–	NS
Yes versus no	1.94 (1.23–3.05)		
Heart failure	NS	0.0148	0.0040
Yes versus no		2.10 (1.16–3.83)	1.97 (1.24–3.13)
Cognitive impairment	NS	–	0.0288
Yes versus no			1.61 (1.05–2.48)
Abnormal saturation	NS	–	0.0175
Yes versus no			1.65 (1.09–2.48)
Site of cancer	–	0.0077	–
Lung cancer versus Other site		2.30 (1.39–3.81)	
Gastrointestinal cancer versus Other site		1.31 (0.75–2.28)	
Urogenital cancer versus Other site		1.25 (0.73–2.15)	
Metastatic cancer^d^	–	< 0.0001	–
Yes versus no		3.53 (2.07–6.01)	
Unknown versus no		1.63 (0.75–3.52)	
Ongoing chemotherapy^e^	–	NS	–

Age, systolic blood pressure, and heart rate are considered as continuous variables. Female gender and Hospitalization ≤ 4 weeks prior to PE and previous venous thromboembolism were not independent predictors of death in all the models, and are not reported in the table

Model I: only included general variables; Model II also included cancer-related variables

COPD, chronic obstructive pulmonary disease

^a^Unknown for 1407 pts

^b^Not assessed in 494 pts

^c^Unknown for 124 pts

^d^Unknown for 104 pts

^e^Unknown for 41

In patients without active cancer, decreasing systolic blood pressure, respiratory rate ≥ 30 breaths/min, abnormal oxygen saturation, abnormal vigilance status at admission, and cognitive impairment were additional independent predictors of death at 30 days.

## Discussion

Our study, specifically dealing with the early management of patients with acute symptomatic PE, shows that since the initial phases of patient management, the risk for death and that for major bleeding are higher in patients with active cancer in comparison to patients with previous cancer or without cancer. In everyday practice, the contemporary clinical management differs in patients with active cancer, previous cancer, or without cancer. In addition, among patients with active cancer, the risk for death due to PE and major bleeding at 30 days from diagnosis of PE differs by type and stage of cancer. Despite cancer is by far the most prevalent cause of death, death due to PE is more common in patients with lung or hematological cancer; patients with brain or hematologic cancer have the highest risk of death due to major bleeding.

The association between cancer and VTE is well known [2]. Our study shows that the most prevalent cancers in patients with acute PE are lung, gastrointestinal, and genitourinary cancers, each accounting for about 20% prevalence. More than 50% of patients with active cancer have metastatic disease at the time of acute PE and the majority are receiving anticancer treatment. These epidemiological data may be of importance when planning future studies as well as the clinical development of anticoagulant agents for the treatment of VTE [15]. In fact, patients with gastrointestinal and genitourinary cancers are prone to bleeding and this makes anticoagulation cumbersome. Still, some international guidelines include warning on the use of oral anticoagulants in these patients, mainly if cancer has not been resected. According to our results, it is conceivable that a not negligible proportion of patients with acute PE will have active cancers with a considerable risk of bleeding (brain, hematological, genitourinary, or gastrointestinal cancers) and this claim for further studies.

The COPE study shows that the higher risk for death and for major bleeding described in patients with active cancer in comparison to patients with previous cancer or without cancer starts in the initial phases of PE management as these risks significantly differ since the in-hospital phase. The risk for death stabilizes after the initial 10 days in patients without cancer, while in patients with active cancer, the risk continues to rise up to 30 days from index PE. These findings were confirmed after adjusting for the severity of acute PE. In fact, in patients with cancer, about 60% of deaths were due to cancer and only 16.5% due to PE.

Our study also shows that the primary site of cancer influences the clinical course of patients since the initial phases of PE. In particular, in patients with lung cancer we observed a very high risk of death and a low risk for major bleeding at 30 days from diagnosis of PE. On the other side, in patients with brain cancer the risk of major bleeding overcomes that of death at 30 days; patients with hematological cancer also had a substantial risk for major bleeding. Despite previous studies addressed the issue of different clinical course of VTE in patients with different types of active cancer, limited data were available on specific cancer types and in the very early phases of VTE [16, 17]. As expected, the overall rate of major bleeding was almost double in patients with active cancer compered to patients with previous cancer or no cancer. The majority of bleedings in patients with active cancer qualified as major due to drop in hemoglobin levels, while the rate of intracranial bleeding was particularly low. Whether this is due to reduced investigations in sick patients remains undefined. Due to small numbers, further analyses on site of cancer and type of major bleeding were not performed. Paucity of data exists concerning patients with VTE and hematological malignancies and this makes difficult to suggest comparisons [19]. Our data obtained in clinical practice with uncontrolled regimens of anticoagulation suggest particular caution in the treatment of patients with brain and hematological cancers until dedicated studies with controlled regimens of anticoagulation will be conducted.

In our study, conducted in a large number of hospitals, the early clinical management of patients with acute symptomatic PE differed based on presence of active cancer, previous cancer, or no cancer. Right ventricle assessment was less commonly obtained in patients with cancer (either active or previous cancer) than in patients with no cancer; thrombolysis was performed more often in patients without cancer, with similar proportion in patients with active cancer and in those with previous cancer. The higher prevalence of contraindications for thrombolysis in patients with active cancer in comparison to the other patient groups could have influenced the numerically lower use of thrombolysis. Parenteral anticoagulants were still used in a substantial proportion of patients with active cancer (over 50% at discharge). These data are in keeping with those from a large international study in patients with VTE [6]. However, a higher proportion of patients with active cancer received DOACs at hospital discharge in the COPE study with respect to the Garfield study. VKAs were almost abandoned in the COPE study, while these agents were used in about 30% of patients in the Garfield registry and in more than 24% of patients with active cancer. Whether this is due to easier DOAC availability across study centers or to increased confidence in DOAC use in the lag time between COPE and Garfield is unknown. In fact, the use of DOACs is now recommended in the international guidelines for the majority of patients with cancer and VTE [8–12]. Overall, our data show that the scenario of anticoagulant treatment is highly heterogeneous and that the availability of DOACs for the treatment of VTE in patients with cancer could implement personalized patient management in clinical practice.

Our study suggests that models for risk stratification in patients with acute symptomatic PE should be personalized based on the presence/absence of active cancer. In fact, in patients with active cancer, cancer-related factors seem to be better predictors of death in comparison to some well-known hemodynamic or respiratory parameters (e.g., systolic blood pressure or respiratory rate). In the COPE study, patients with active cancer had worse clinical outcome in comparison to patients with previous cancer or no cancer despite a higher prevalence of intermediate–low-risk PE according to the ESC stratification model. This is easily explained by the high risk of death due to cancer in patients with active cancer. Overall, these findings renew the issue of tailoring decision-making for patient management in clinical practice based on both, the overall risk for death and the risk for death attributable to PE [20].

Our study has some limits. Despite the large sample size and the good representativeness of the three groups of patients with active cancer, previous cancer, and no cancer, some subgroups of patients with specific sites of primary cancers are under-represented. Moreover, as data on type of chemotherapy and site of metastatic disease were not collected, the role of these figures on the course of PE was not valuable. In addition, COPE is a non-intervention study, and this makes comparisons between treatment strategies as hypothesis generating only. However, the large number of included patients allows powered analyses in several contexts.

However, our study has also some strengths. The inclusion of more than 5000 patients makes COPE the largest registry ever in patients with acute PE. The large number of study sites all over Italy regardless of academic or non-academic nature as well as the inclusion of study sites with different specialties (cardiology, internal medicine, and emergency medicine) should have guaranteed the representativeness of the study sample.

### Interpretation

In conclusion, among patients with acute symptomatic PE, those with active cancer have a high risk for death and major bleeding since the initial days after diagnosis of PE. The risk for death and major bleeding varies in patients with acute PE based on primary site of active cancer. Our findings may challenge clinical practice and inform future studies on the treatment of patients with acute PE.

### Supplementary Information

Below is the link to the electronic supplementary material.

Supplementary file 1 (DOCX 33 KB)

## Data Availability

Study data will be available starting at least six months after publication after formal request to the corresponding author, and after approval by the Steering Committe of the study.

## Abbreviations

DOAC: Direct oral anticoagulants
PE: Pulmonary embolism
VTE: Venous thromboembolism
RG: Reference group
