# Correction: Acute pulmonary embolism and cancer: findings from the COPE study

**DOI:** 10.1007/s00392-023-02369-z

**Published:** 2024-01-22

**Authors:** Cecilia Becattini, Ludovica Anna Cimini, Giorgio Bassanelli, Aldo P. Maggioni, Fulvio Pomero, Ilaria Lobascio, Iolanda Enea, Daniela P. Pomata, Maria Pia Ruggieri, Beniamino Zalunardo, Anna Novelli, Stefania Angela Di Fusco, Marco Triggiani, Marco Marzolo, Chiara Fioravanti, Giancarlo Agnelli, Lucio Gonzini, Michele M. Gulizia, on behalf of COPE Investigators

**Affiliations:** 1 Internal, Vascular and Emergency Medicine-Stroke Unit, University of Perugia, Piazzale Lucio Severi 1, 06129 Perugia, Italy; 2 Department of Cardiology, Ospedale Alessandro Manzoni, Lecco, Italy; 3 ANMCO Research Center, Heart Care Foundation, Florence, Italy; 4 Department of Internal Medicine, Ospedale Michele E Pietro Ferrero, Verduno, Italy; 5 Department of Cardiology, Ospedale Civile, Arzignano, Italy; 6 U.O.C. Medicina e Chirurgia d’Urgenza, A.O.R.N. “S. Anna e S. Sebastiano”, Caserta, Italy; 7 Medicina d’Urgenza E Pronto Soccorso, Ospedale Policlinico S. Orsola-Malpighi, Bologna, Italy; 8 U.O.C. Medicina d’Urgenza E Pronto Soccorso, AO San Giovanni Addolorata, Rome, Italy; 9 Angiology Unit, Azienda ULSS 2 Marca Trevigiana, Castelfranco Veneto, Treviso, Italy; 10 Pronto Soccorso E Medicina d’Urgenza, Ospedali Riuniti, Leghorn, Italy; 11 Cardiologia Clinica E Riabilitativa, P.O. San Filippo Neri, Rome, Italy; 12 U.O. Cardiologia, Ospedale Civile “La Memoria”, Gavardo, Brescia, Italy; 13 U.O.C. Medicina Interna-Angiologia, Ospedale S. Maria Della Misericordia, Rovigo, Italy; 14 Medicina Interna, Ospedali Galliera, Genoa, Italy; 15 Division of Cardiology, Garibaldi-Nesima Hospital, Catania, Italy

**Correction: Clinical Research in Cardiology**

10.1007/s00392-023-02323-z

The author put the list of contributors into the supplementary material 2 instead of mention it in the text.

The article has now been corrected.

## Appendix

**Steering Committee**

Cecilia Becattini (Chairperson), Michele M. Gulizia (Co-Chair), Giancarlo Agnelli, Francesco Dentali, Andrea Di Lenarda, Iolanda Enea, Andrea Fabbri, Aldo P. Maggioni, Fulvio Pomero, Maria Pia Ruggeri.

**Executive Committee**

Cecilia Becattini, Donata Lucci, Aldo P. Maggioni.

**Clinical Event Adjudication Committee**

Maria Cristina Vedovati (Chairman), Michele Duranti, Francesco Guercini, Palo Groff, Melina Verso, Gianna Fabbri (coordinator), Martina Savoia (secretary).

**Coordinating Center**

ANMCO Research Center, Fondazione per il Tuo cuore, Florence, Italy (Donata Lucci, Ester Baldini, Barbara Bartolomei Mecatti, Francesca Bianchini, Martina Ceseri, Gianna Fabbri, Lucio Gonzini, Marco Gorini, Andrea Lorimer, Giampietro Orsini, Martina Tricoli).

**Participating Centers and Investigators**

Perugia, A.O. Perugia, Medicina Interna Vascolare e d'Urgenza (LA Cimini, C Becattini, G Agnelli, V Cesarini); Lido di Camaiore, Nuovo Osp. Versilia, Medicina d'Urgenza e PS (M Sanna, G Pepe, C Marchetti, M Olivan Roldan); Rovereto, Osp. S.M. del Carmine, Medicina Interna (L Lenzi, S Cozzio, P Tomio); Treviso, Osp. Ca' Foncello, PS Medicina d'Urgenza (M Diamanti, A Beltrame, L Glinski, M Treleani); Empoli, Osp. San Giuseppe, Medicina d'Urgenza (A Coppa, S Vanni, P Bartalucci, A Taccone); Pordenone, Osp. di Pordenone, PS e Medicina d'Urgenza (C Costacurta, P Bortolotti, M Bortolussi); Bergamo, ASST Papa Giovanni XXIII, Centro EAS (M De Vecchi, F Zanardi, I Greco, R Cosentini); Trieste, ASUGI - Polo Cardiol. Cattinara, Medicina d'Urgenza e PS (R Gerloni, N Artusi, F Cominotto, UG Sisto); Rovigo, Osp. S.M. della Misericordia, UOC Cardiologia (C Picariello, L Roncon, A Maddalozzo); Ancona, Osp. Riuniti, SOD PS e Medicina d'Urgenza (C Nitti, F Riccomi, M Buzzo); Lecco, Osp. A. Manzoni, SC Cardiologia (G Bassanelli, S Savonitto, A Bianchi); Arzignano, Osp. Civile, UOC Cardiologia (C Bilato, I Lobascio, C Dalla Valle); Bologna, Osp. Policlinico S. Orsola-Malpighi, UOC Medicina d'Urgenza e PS (DP Pomata, F Giostra, AL Tinuper); Castelfranco Veneto, Stabilimento Ospedaliero di Castelfranco, UOC Angiologia (B Zalunardo, A Visonà, C Panzavolta); Livorno, Osp. Riuniti, PS e Medicina d'Urgenza (A Novelli, A Bertini, C Granai); Milano, ASST Osp. Metropolitano Niguarda, Medicina d'Urgenza e PS (S Colombo, E Periti, L Bonacchini); Erice, P.O. S. Antonio Abate di Trapani, UOC Cardiologia e UTIC (MG Abrignani, MF Casciolo, A D'Amato); Roma, Osp. Santo Spirito, UOC Cardiologia (AB Scardovi, R Ricci, S Iosi); Pavullo nel Frignano, Presidio Ospedaliero, Divisione Medicina Generale (MC Fontana, A Marrazzo); Grosseto, Osp. della Misericordia, Pronto Soccorso (M Borselli); Roma, P.O. San Filippo Neri - ASL Roma 1, Cardiologia Clinica e Riabilitativa (SA Di Fusco, F Colivicchi); Caserta, A.O. S. Anna e S. Sebastiano, UOC Medicina e Chirurgia d'Urgenza (I Enea); Gavardo, Osp. Civile 'La Memoria', U.O. Cardiologia (M Triggiani, I Papa, GF Pasini); Genova, E.O. Ospedali Galliera, Medicina Interna (C Fioravanti, S Panarello, F Raggi); Rovigo, Osp. S.M. della Misericordia, UOC Medicina Interna – Angiologia (M Marzolo, S Cuppini, M Milan); Sassuolo, Osp. di Sassuolo, Medicina d'Urgenza (M Barchetti); Bari, Osp. San Paolo, Cardiologia-UTIC (D De Laura, P Caldarola); Fidenza, Osp. Civile, PS e Medicina d'Urgenza (R Fiorini, G Rastelli); Genova, Osp. Policlinico San Martino, UO Cardiologia (P Ameri, G La Malfa); Treviglio, Osp. Treviglio-Caravaggio, Cardiologia (F Cinelli, PC Sganzerla); Sanremo, Presidio Ospedaliero, UO Cardiologia (S Ubaldi, FA Sanchez); Brescia, Istituto Osp. Fondazione Poliambulanza, UO Cardiologia (C Forgione, C Cuccia); Faenza, Osp. per gli Infermi, Pronto Soccorso (S Predieri, S Fusco); Magenta, Osp. Civile Fornaroli, Medicina Interna (N Mumoli, C Porta); Poggibonsi, Osp. dell'Alta Val d'Elsa, UOSD Cardiologia-UTIC (M Romei, M Lucidi); Roma, Osp. S. Andrea di Roma, UOC Cardiologia (A Romaniello, M Volpe); Chieri, Osp. Maggiore, SC Medicina Interna (P Mogni, E Pizzolato); Fermo, Osp. Civile A. Murri, Medicina Interna (GP Martino, G Bitti); Modena, Osp. Civile di Baggiovara, Medicina d'Urgenza e PS (G Righini, G Bandiera); Potenza, A.O. San Carlo, UOC PS Accettazione Medicina d'Urgenza (E Pennacchio, S Limauro); Brindisi, Osp. Perrino, UOC Cardiologia (A Dachille, G Ignone); Cosenza, Osp. Annunziata, UOC Cardiologia (GF Fuscaldo, FM De Rosa); Scandiano, Osp. C. Magati, AUSL - IRCCS di Reggio Emilia, Divisione Medicina Interna (N Vazzana, G Chesi); Ravenna, Osp. Civile S.M. delle Croci, PS e Medicina d'Urgenza (F Di Filippo, L Pierpaoli); Catanzaro, Osp. Civile Pugliese, UTIC-Emodinamica e Card. Interventistica (A Corapi, M Vatrano); Firenze, Osp. San Giovanni di Dio, Medicina Interna (C Angotti, F Baccetti); Milano, Multimedica Osp. San Giuseppe, Pneumologia (SA Harari, F Luisi); Prato, Osp. Santo Stefano, PS e Medicina d'Urgenza (E Daghini, E De Curtis); Reggio Calabria, Grande Osp. Metropol.-Bianchi Melacrino, Divisione Cardiologia (F Lucà, F Ciancia); Vittoria, Osp. Riccardo Guzzardi, UOC Cardiologia-UTIC (S Blandizzi, GV Lettica); Cortona, Osp. Valdichiana S. Margherita, UOS Cardiologia e HDU (S D'Orazio, F Cosmi); Bologna, Osp. Maggiore, Medicina Interna A (S Zaccaroni, M Silingardi); Roma, Osp. Sandro Pertini, Medicina d'Urgenza (V Valeriano, FR Pugliese); Sassari, Osp. SS. Annunziata, PS e Medicina d'Urgenza (AP Murgia, P Pinna Parpaglia); Città di Castello, P.O. Città di Castello, Medicina Interna (L Martinelli, C Caponi); Matera, Osp. Madonna delle Grazie, UO Cardiologia UTIC (MA Clemente, A Ciccarone); Milano, ASST Santi Paolo e Carlo – P.O. San Carlo, Cardiologia-UCC (A Bongarzoni, M Garagiola); Reggio Emilia, P.O. Santa Maria Nuova - AUSL RE IRCCS, Medicina Cardiovascolare (MC Leone, MR Veropalumbo); Napoli, A.O.R.N. Cardarelli, Medicina d'Urgenza (M Sacco, P Morella); Trento, Osp. Santa Chiara, Medicina Interna I (S Dorigoni, D Peterlana); Catania, P.O. Garibaldi Centro, UOC Cardiologia (R Di Paola, S Felis); Foggia, Osp. Riuniti, SC Cardiologia Univ.-UTIC (M Correale, ND Brunetti); San Benedetto del Tronto, Osp. Madonna del Soccorso, Medicina d'Urgenza (G Petrelli, FT Feliziani); Avezzano, Osp. Civile SS. Filippo e Nicola, UOSD Angiologia (D Mastroiacovo, R Romualdi); Casalmaggiore, Osp. Civile Oglio Po, Medicina Generale (F Pasin, S Bonardi); Monza, Policlinico di Monza, Cardiologia Clinica e Scompenso Cardiaco (P Delfino, C Scifo); Pavia, Fondazione IRCCS Policlinico S. Matteo, Pronto Soccorso (G Savioli, IF Ceresa); Forlì, Osp. G.B. Morgagni - L. Pierantoni, U.O. Medicina d'Urgenza-PS (M Galeotalanza, B Benazzi); Milano, Osp. Maggiore Policlinico, PS e Medicina d'Urgenza (M Porzio, F Rosini); Roma, A.O. San Giovanni Addolorata, UOC PS-Breve Osservazione (C Ancona, C Verrelli); Verona, A.O.U.I. di Verona, Medicina Gen. Malattie Aterotrombotiche (A Fratta Pasini, L Dalle Carbonare); Bari, A.O.U. Policlinico, UO Cardiologia Universitaria (N Bozza, F Nacci); Catania, A.O. Cannizzaro, UOC Cardiologia con UTIC ed Emodinamica (MA Scarabelli, F Amico); Legnano, ASST Ovest Milanese – P.O. di Legnano, Medicina Generale (C Marchesi, A Mazzone); Manduria, Osp. Civile M. Giannuzzi, Cardiologia e UTIC (R Di Tommaso, F Cocco); Modena, Osp. Policlinico, Pronto Soccorso (G Pezzuto, A Luciani); Noventa Vicentina, Ospedale Civile, Medicina Generale 2 (P Zamboni, M Muriago); Pontedera, Osp. Generale Provinciale Lotti, UO Malattie Cardiovascolari VDE e AVC (L Del Pesce, AR Lucarini); Roma, Osp. San Camillo, Medicina d'Urgenza e Cure Differenziate (E Guglielmelli); Firenze, Osp. S.M. Nuova, Medicina A2 (V Vannucchi, F Moroni); Catania, A.O. Cannizzaro, Medicina Interna (D Fichera, L Malatino); Catania, P.O. Garibaldi Centro, Medicina Interna Area Critica (C Sgroi, IM Morana); Palermo, Osp. Buccheri La Ferla FBF, Dip. Emergenza, Accettaz. e dei Servizi (S Cicero, D La Rosa); Rivoli, Osp. degli Infermi, Medicina Generale (F Mete, M Gino); Massa Marittima, Osp. S. Andrea - Alta Maremma, Divisione Medicina (A De Palma, M Alessandri); Pescia, Osp. SS. Cosma e Damiano, Medicina Generale (V Maestripieri); Bassano del Grappa, Osp. San Bassiano, SC Medicina Interna (M Battocchio); Città di Castello, P.O. Città di Castello, UO Cardiologia (MT De Santis); Cittadella, Osp. di Cittadella, UO Cardiologia (F Saladini); Monza, Osp. San Gerardo, UO Cardiologia-UTIC (DC Corsi); Reggio Emilia, P.O. Santa Maria Nuova - AUSL RE IRCCS, Medicina d'Urgenza e PS (N Macarone Palmieri); Cremona, Osp. di Cremona, Medicina Interna (M Giorgi Pierfranceschi); Imola, Osp. Nuovo S.M. della Scaletta, Medicina d'Urgenza e PS (V Palmonari); Napoli, Osp. Buon Consiglio FBF, SC Medicina (L Fontanella); Novara, A.O.U. Maggiore della Carità, Cardiologia II (L Airoldi); Palermo, A.O.R. Villa Sofia-Cervello P.O. Villa Sofia, UOC Cardiologia e UTIC - Villa Sofia (M Bonocore); Todi, Nuovo Osp. Media Valle del Tevere, Divisione Medicina Interna (U Paliani); Udine, P.O.U. S.M. della Misericordia, PS Medicina d'Urgenza (L Iogna Prat); Arzignano, Osp. Civile, Medicina Interna (L Chiecchi); Bari-Carbonara, Osp. di Venere, UOC Cardiologia (M Cuonzo); Bergamo, ASST Papa Giovanni XXIII, Divisione Medicina Interna (A Paludo); Milano, ASST Santi Paolo e Carlo – P.O. San Carlo, PS e Medicina d'Urgenza (D Padula); Sesto San Giovanni, IRCCS Policlinico Multimedica, UO Cardiologia (A Antonelli); Siena, A.O.U Senese Osp. S.M. alle Scotte, UOC Medicina Interna 2 (M Bicchi); Bagno a Ripoli, Osp. S.M. Annunziata, DEA (G Tota); Castel Volturno, Pineta Grande Hospital, Medicina d'Urgenza (M Ariello); Gallipoli, Osp. Civile Sacro Cuore di Gesù, UO Cardiologia UTIC (R Sai); Pinerolo, Osp. Civile E. Agnelli, Medicina-Chirurgia Accettazione-Urgenza (M Civita); Teramo, Osp. Civile G. Mazzini, UOC Pronto Soccorso (M Tucci); Vicenza, Osp. Civile San Bortolo, UOC Cardiologia (G Barbati); Asti, Osp. Cardinal Massaia, Medicina A (M Conti); Augusta, Osp. E. Muscatello, UO Cardiologia – UTIC (R Cettina); Genova, Osp. Policlinico San Martino, Medicina d'Urgenza 1 (O Magnani); Lamezia Terme, Osp. Giovanni Paolo II, UO Cardiologia e UTIC (M Levato); Luino, Osp. di Luino, Medicina Generale (V Gessi); Montebelluna, Osp. San Valentino, Medicina Interna (M De Rui); Piombino, Osp. Villamarina, Medicina Generale (A Bellizzi); Rimini, Osp. Infermi, UO Cardiologia (L Farneti); Alba, Osp. Civile San Lazzaro, Medicina Generale (P Salomone); Bari, A.O.U. Policlinico, UOC Cardiologia Ospedaliera (A Mannarini); Empoli, Osp. San Giuseppe, Medicina Interna II (E Grifoni); Livorno, Ospedali Riuniti, Medicina Generale (S Del Ghianda); Milano, Centro Cardiologico Monzino, UO Terapia Intensiva Cardiologica (UTIC) (J Campodonico); Osio Sotto, I.O.B. - Policlinico San Marco, UO Cardiologia (N De Cesare); Pesaro, P.O. San Salvatore, Cardiologia e UTIC (D Mutone); San Bonifacio, Osp. G. Fracastoro, UOC Cardiologia (P Pasoli); Saronno, P.O. di Saronno, UOC Cardiologia (S Meloni); Agrigento, Osp. San Giovanni di Dio, UO Cardiologia e UTIC (A Frenda); Milano, ASST Osp. Metropolitano Niguarda, Cardiologia 1 – Emodinamica (G Viola); Roma, Policlinico Umberto Primo, Cardiologia B - Cardiologia e Angiologia (C Torromeo); San Fermo della Battaglia, Osp. S. Anna, UOC Cardiologia (C Campana); Savona, Osp. San Paolo, Medicina Interna e Cure Intermedie (MC Pistone); Milano, Osp. San Luca - Istituto Auxologico, UO Cardiologia (S Caravita); Potenza, A.O. San Carlo, SSD UTIC (CP La Creta); San Severo, P.O. T. Masselli Mascia, SC Cardiologia – UTIC - Riabil. Card. (F Miscio); Siracusa, Osp. Umberto I, Div. Medicina - Centro Ipertensione (M Loreno); Cecina, Osp. Civile Bassa Val di Cecina, Medicina Generale (P Fenu); Montevarchi, Osp. Valdarno 'S.M. della Gruccia', Medicina Interna (M Mazzetti); Pescia, Osp. SS. Cosma e Damiano, UO Cardiologia – UTIC (D Rossini); Genova, Osp. P.A. Micone, SC Cardiologia – UTIC (M Brunacci); Melfi, Osp. S. Giovanni di Dio, UO Cardiologia e UTIC (A Capuano); Pozzuoli, Osp. S.M. delle Grazie, UO Cardiologia – UTIC (G Tagliamonte); Savigliano, Osp. Maggiore SS. Annunziata, Medicina e Chirurgia d'Urgenza (M Pinelli); Torino, Osp. Maria Vittoria, UO Cardiologia (F Ballocca); Arezzo, Osp. San Donato, Medicina Interna (G Parca); Lodi, P.O. di Lodi, Medicina Generale (SM Pasini); Matera, Osp. Madonna delle Grazie, Pronto Soccorso TSI (MG Maragno); Sassuolo, Osp. di Sassuolo, Medicina Interna (F Vecchi); Ancona, Istituto Scientifico INRCA, UO Cardiologia/UTIC/Telecardiologia (L Mancinelli); Ariano Irpino, P.O. Sant'Ottone Frangipane, Medicina Generale (A Cavalli); Figline Valdarno, P.O. Serristori, Medicina Interna e d'Urgenza (F Di Mare); Forlì, Osp. G.B. Morgagni - L. Pierantoni, UO Cardiologia (E Conficoni); Genova, E.O. Ospedali Galliera, SC Cardiologia (R Miceli); Pordenone, Osp. di Pordenone, SOC Cardiologia (R Pecoraro); Torino, Osp. Humanitas Gradenigo, Medicina e Chirurgia d'Urgenza (C Fonti); Varese, Osp. di Circolo e Fondazione Macchi, Medicina Generale I (S Pegoraro); Casarano, P.O. F. Ferrari, Cardiologia – UTIC (GC Piccinni); Catania, Policlinico Catania P.O. G. Rodolico, Cardiologia UTIC (G Caruso); Modena, Osp. Policlinico, UO Cardiologia (G Boriani); Pescara, Osp. Civile dello Spirito Santo, UO Geriatria (P Lanzillotta); Roma, Osp. Fatebenefratelli, Medicina Interna (P Piccolo); Roma, Policlinico Casilino, UO Cardiologia (L Calò); Trieste, ASUGI - Polo Cardiologico Cattinara, SOC Cardiologia (D Stolfo); Avellino, A.O.R.N. San Giuseppe Moscati, Medicina Interna (S Mangiacapra); Fermo, Osp. Civile A. Murri, UO Cardiologia (A Marziali); Frosinone, Osp. Fabrizio Spaziani, Cardiologia-UTIC (MC Volponi); La Spezia, Osp. S. Andrea, Medicina Interna (G Querci); Vercelli, Osp. S. Andrea, Medicina Interna (R Terribile); Vignola, Osp. Civile, UO Pronto Soccorso (M Menabue); Viterbo, Osp. Belcolle, Medicina Generale (A Fiorentini); Andria, Osp. L. Bonomo, Cardiologia – UTIC (RL Musci); Nuoro, Osp. San Francesco, Cardiologia – UTIC (S Uras); Roma, Osp. San Camillo, UOC Cardiologia (MP Cicini); Varese, Osp. di Circolo e Fondazione Macchi, Geriatria (S Pegoraro); Bibbiena, Osp. Civile, Medicina Interna (S Manetti); Catania, P.O. Garibaldi-Nesima - ARNAS Garibaldi, UOC Cardiologia (GM Francese); Cuneo, A.O. Santa Croce e Carle, Divisione Medicina Interna (R Melchio); Lanciano, Osp. 'Renzetti', Medicina Interna (N Scorpiglione); Lido di Camaiore, Nuovo Osp. Versilia, Medicina Generale (D Carrara); Milano, ASST Osp. Metropolitano Niguarda, Medicina Interna 1 (A Pani); Napoli, A.O.U. Federico II, UOC Cardiologia e UTIC (C Morisco); Vibo Valentia, Osp. Civile G. Jazzolino, Medicina d'Urgenza (M Rodolico).

