# Supplementary material for: Acute pulmonary embolism and cancer: findings from the COPE study
Source: Clin Res Cardiol. 2023 Nov 15;113(2):288–300. doi: 10.1007/s00392-023-02323-z (PMC10850192; doi:10.1007/s00392-023-02323-z)
Supplement: Supplementary file 1 — (DOCX 33 KB) [file 392_2023_2323_MOESM1_ESM.docx]

**Supplementary material**

**Supplementary Table I.** Clinical presentation of PE in the total population and by cancer status

|  | **Total**  **population**  **(N=4956)** |  | **Active cancer**  **(n=832)** | | **Previous**  **cancer**  **(n=464)** | | **No cancer**  **(n=3660)** | **P** | |  |
| --- | --- | --- | --- | --- | --- | --- | --- | --- | --- | --- |
| Days from symptom onset to diagnosis | 4±8 |  | | 4±7 | | 4±9 | 4±8 | | 0.38 | |
| Dyspnea, n (%) | 3939 (79.5) |  | 681 (81.9) | | 374 (80.6) | | 2884 (78.8) | 0.12 | |  |
| Chest pain, n (%)  Pleuritic (1431 pts)ª  Angina-like (1431 pts) ª | 1477 (29.8)  887 (62.0)  544 (38.0) |  | 183 (22.0)  105 (60.0)  70 (40.0) | | 111 (23.9)  64 (60.4)  42 (39.6) | | 1183 (32.3)  718 (62.4)  432 (37.6) | <0.0001 | |  |
|  |  |  |  |  |  |  |  | 0.78 | |  |
| Tachicardia, n (%) | 702 (14.2) |  | 140 (16.8) | | 57 (12.3) | | 505 (13.8) | 0.04 | |  |
| Haemoftoe, n (%) | 89 (1.8) |  | 15 (1.8) | | 6 (1.3) | | 68 (1.9) | 0.69 | |  |
| Syncope, n (%) | 599 (12.1) |  | 59 (7.1) | | 79 (17.0) | | 461 (12.6) | <0.0001 | |  |
| Lypothimia (4602 pts), n (%) ª | 422 (9.1) |  | 65 (8.5) | | 50 (11.6) | | 307 (9.0) | 0.18 | |  |
| Lower limb edema, n (%) | 1464 (29.5) |  | 224 (26.9) | | 106 (22.8) | | 1134 (31.0) | 0.0003 | |  |

ª Percentages were evaluated on patients with data available reported in brackets for each variable

**Supplementary Table II.** Test performed in the total population and by cancer status

| **Test performed** | **Total**  **population**  **(N=4956)** | **Active cancer**  **(n=832)** | **Previous**  **cancer**  **(n=464)** | **No cancer**  **(n=3660)** | **P** |
| --- | --- | --- | --- | --- | --- |
| **Initial tests** | | | | |  |
| D-dimer, n (%)  *Increased, n (%)* | 3838 (77.4)  3762 (98.0) | 491 (59.0)  481 (98.0) | 353 (76.1)  345 (97.7) | 2994 (84.8)  2936 (98.1) | <0.0001  0.74 |
| Chest X ray, n (%)  *Abnormal, n (%)* | 2692 (54.3)  1313 (48.8) | 371 (44.6)  229 (61.7) | 272 (58.6)  129 (47.4) | 2049 (56.0)  955 (46.6) | <0.0001  <0.0001 |
| Blood gas analysis, n (%) | 4068 (82.1) | 666 (80.1) | 392 (84.5) | 3010 (82.2) | 0.12 |
| EKG, n (%)  *Abnormal, n (%)* | 4804 (96.9)  1444 (30.1) | 793 (95.3)  200 (25.2) | 449 (96.8)  156 (34.7) | 3562 (97.3)  1088 (30.5) | 0.01  0.001 |
| **Imaging** | | | | |  |
| CT angiography, n (%)  *Positive for PE, n (%)*  *More proximal localization of emboli:*  *Main pulmonary arteries, n (%)*  *Segmental, n (%)*  *Isolated subsegmental, n (%)*  *Bilateral emboli, n (%)*  *CT assessment of the RV, n (%)* | 4782 (96.5)  4772 (99.8)  2093 (43.9)  1252 (26.2)  215 (4.5)  1212 (25.4)  545 (11.4) | 798 (95.9)  796 (99.8)  327 (41.1)  246 (30.9)  24 (3.0)  199 (25.0)  68 (8.5) | 449 (97.0)  447 (99.6)  183 (40.9)  120 (26.9)  20 (4.5)  124 (27.7)  35 (7.8) | 3535 (96.6)  3529 (99.8)  1583 (44.9)  886 (25.1)  171 (4.9)  889 (25.2)  442 (12.5) | 0.61  0.47  0.06  0.003  0.08  0.49  0.0002 |
| Lower limb ultrasonography, n (%)  *Positive for DVT, n (%)*  *More proximal localization:*  *Inferior vena cava, n (%)*  *Isolated Distal, n (%)* | 3899 (78.7)  2386 (61.2)  28 (1.2)  312 (13.1) | 577 (69.4)  393 (68.1)  7 (1.8)  56 (14.3) | 373 (80.4)  222 (59.5)  4 (1.8)  41 (18.5) | 2949 (80.6)  1771 (60.1)  17 (1.0)  215 (12.1) | <0.0001  0.001  0.26  0.02 |
| Echocardiography, n (%)  *Abnormal*, n (%)* | 4011 (80.9)  2424 (60.4) | 604 (72.6)  346 (57.3) | 369 (79.5)  256 (69.4) | 3038 (83.0)  1822 (60.0) | <0.0001  0.0005 |
| **Biomarkers** | | | | |  |
| Troponin, n (%)  *Abnormal, n (%)* | 4122 (83.2)  2261 (54.9) | 615 (73.9)  331 (53.8) | 396 (85.3)  232 (58.6) | 3111 (85.0)  1698 (54.6) | <0.0001  0.27 |
| BNP, n (%)  *Abnormal, n (%)* | 1146 (23.1)  583 (50.9) | 167 (20.1)  82 (49.1) | 129 (27.8)  72 (55.8) | 850 (23.2)  429 (50.5) | 0.006  0.47 |

EKG, electrocardiogram; NMR, Nuclear Magnetic Resonance; DVT, Deep Vein Thrombosis;

*echocardiography was obtained within 24 hours from admission in 2030 patients (41%) and within 48 hours from admission in 3150 patients (64%).

**Supplementary Table III** Risk for death according to ESC guidelines, by cancer status

|  | **Total population**  **(n= 4956)** | **Cancer status** | | |
| --- | --- | --- | --- | --- |
|  |  | **Active**  **(n 832)** | **Previous**  **(n 464)** | **No cancer**  **(n 3660)** |
| Low risk  Intermediate  Low  High  Unknown  High risk  Unknown | 1626 (32.8)  3117 (62.9)  1780 (35.9)  1132 (22.8)  205 (4.1)  171 (3.4)  42 (0.9) | 0 (0.0)  804 (96.6)  **528 (63.4)**  181 (21.8)  95 (11.4)  28 (3.4)  0 (0.0) | 138 (29.7)  303 (65.3)  147 (31.7)  140 (30.2)  16 (3.5)  17 (3.7)  6 (1.3) | 1488 (40.7)  2010 (54.9)  1105 (30.2)  811 (22.2)  94 (2.6)  126 (3.4)  36 (1.0) |

**Supplementary Table IV.** Use of anticoagulant agents during hospital stay and at discharge in patients with active cancer, by site of cancer

|  | **Active cancer**  **(n=832)** | **Primary site of cancer** | | | | | | |
| --- | --- | --- | --- | --- | --- | --- | --- | --- |
|  |  | **Lung**  **(n=165)** | **GI**  **(n=175)** | **GU**  **(n=191)** | **Hematological**  **(106)** | **Brain**  **(n=35)** | **Other**  **(n=142)** | **Unknown**  **(n=18)** |
| **Anticoagulant during hospital stay** | | | | | | | | |
| *≥1 parenteral anticoagulant°, n (%)* | 787 (94.6) | 154 (93.3) | 169 (96.6) | 181 (94.8) | 99 (93.4) | 34 (97.1) | 134 (94.4) | 16 (88.9) |
| DOACs, n (%) | 214 (25.7) | 39 (23.6) | 36 (20.6) | 61 (31.9) | 28 (26.4) | 9 (25.7) | 37 (26.1) | 4 (22.2) |
| VKAs, n (%) | 18 (2.2) | 3 (1.8) | 4 (2.3) | 2 (1.1) | 7 (6.6) | 0 (0.0) | 2 (1.4) | 0 (0.0) |
| **Revascularization during hospital stay** | | | | | | | | |
| Thrombolysis, n (%)  *percutaneous, n (%)*  *systemic, n (%)*  Contraindication for thrombolysis, n (%) | 28 (3.4)  *5 (17.9)*  *22 (78.6)*  156 (18.8) | 4 (2.4)  2 (50.0)  2 (50.0)  33 (20.0) | 1 (0.6)  0 (0.0)  1 (100)  38 (21.7) | 8 (4.2)  0 (0.0)  7 (87.5)  26 (13.6) | 10 (9.4)  1 (10.0)  8 (80.0)  14 (13.2) | 1 (2.9)  1 (100)  0 (0.0)  17 (48.6) | 2 (1.4)  1 (50.0)  2 (100)  23 (16.2) | 2 (11.1)  0 (0.0)  2 (100)  5 (27.8) |
| Vena cava filter, n (%) | 17 (2.0) | 1 (0.6) | 2 (1.1) | 4 (2.1) | 2 (1.9) | 2 (5.7) | 5 (3.5) | 1 (5.6) |
| **Anticoagulants at discharge*** | | | | | | | | |
|  | **Active cancer**  **(n=706)** | **Lung**  **(n=135)** | **GI**  **(n=148)** | **GU**  **(n=167)** | **Hematological**  **(82)** | **Brain**  **(n=32)** | **Other**  **(n=127)** | **Unknown**  **(n=15)** |
| Parenteral agents, n (%) | 385 (54.5) | 80 (59.3) | 92 (62.2) | 80 (47.9) | 39 (47.6) | 22 (68.8) | 62 (48.8) | 10 (66.7) |
| DOACs, n (%) | 304 (43.1) | 52 (38.5) | 54 (36.5) | 84 (50.3) | 37 (45.1) | 10 (31.3) | 62 (48.8) | 5 (33.3) |
| VKAs, n (%) | 18 (2.6) | 3 (2.2) | 3 (2.0) | 3 (1.8) | 6 (7.3) | 0 (0.0) | 3 (2.4) | 0 (0.0) |
| Oral agents, n (%) | 322 (45.6) | 55 (40.7) | 57 (38.5) | 87 (52.1) | 43 (52.4) | 10 (31.3) | 65 (51.2) | 5 (33.3) |

**among 4555 patients discharged alive from hospital within 30 from EP diagnosis*

*°use of variable sequences of UFH, LMWH and/or fondaparinux in every single patient was reported*

**Supplementary Table V** Cause of death in patients with active cancer by cancer features

|  | Active cancer  (n= 832) | Cause of death at 30 days | | | | | | |
| --- | --- | --- | --- | --- | --- | --- | --- | --- |
|  |  | Death | Death due to PE | Death due to cancer | CV non PE | Major bleeding | Other non CV | Unknown cause |
| Primary site of cancer |  | | | | | | | |
| Lung, n (%) | 165 (19.8) | 37/165 (22.4) | 7/165 (4.2) | 25/165 (15.2) | 3/165 (1.8) | 0/165 (0.0) | 1/165 (0.6) | 1/165 (0.6) |
| Gastrointestinal, n (%) | 175 (21.0) | 24/175 (13.7) | 3/175 (1.7) | 16/175 (9.1) | 0/175 (0.0) | 1/175 (0.6) | 2/175 (1.1) | 2/175 (1.1) |
| Urogenital, n (%) | 191 (23.0) | 25/191 (13.1) | 3/191 (1.6) | 15/191 (7.9) | 0/191 (0.0) | 1/191 (0.5) | 5/191 (2.6) | 1/191 (0.5) |
| Hematological, n (%) | 106 (12.7) | 12/106 (11.3) | 3/106 (2.8) | 0/106 (0.0) | 2/106 (1.9) | 3/106 (2.8) | 4/106 (3.8) | 0/106 (0.0) |
| Brain, n (%) | 35 (4.2) | 2/35 (5.7) | 0/35 (0.0) | 1/35 (2.9) | 0/35 (0.0) | 1/35 (2.9) | 0/35 (0.0) | 0/35 (0.0) |
| Other, n (%) | 142 (17.1) | 13/142 (9.2) | 2/142 (1.4) | 8/142 (5.6) | 0/142 (0.0) | 0/142 (0.0) | 2/142 (1.4) | 1/142 (0.7) |
| Not known, n (%) | 18 (2.2) | 2/18 (11.1) | 1/18 (5.6) | 1/18 5.6) | 0/18 (0.0) | 0/18 (0.0) | 0/18 (0.0) | 0/18 (0.0) |
| Metastatic (728 pts)^a^, n (%) | 419 (57.6) | 87/419 (20.8) | 10/419 (2.4) | 62/419 (14.8) | 2/419 (0.5) | 3/419 (0.7) | 8/419 (1.9) | 2/419 (0.5) |
| Ongoing chemotherapy (791 pts)^a^, n (%) | 364 (46.0) | 52/364 (14.3) | 6/364 (1.7) | 31/364 (8.5) | 2/364 (0.6) | 4/364 (1.1) | 9/364 (2.5) | 0/364 (0.0) |
| Ongoing radiotherapy (788 pts)^a^, n (%) | 128 (16.2) | 19/128 (14.8) | 1/128 (0.8) | 12/128 (9.4) | 1/128 (0.8) | 3/128 (2.3) | 9/128 (7.0) | 0/128 (0.0) |

^a^Percentages were evaluated on patients with data available reported in brackets for each variable

**Supplementary Table VI** Type of major bleeding in patients with active cancer by site of cancer

|  | **Active cancer**  **(n= 832)** | **Study outcomes at 30 days** | | | | | | | |
| --- | --- | --- | --- | --- | --- | --- | --- | --- | --- |
|  |  | **Major bleeding** | **Fatal MB°** | **ICH** | **Hb drop ≥ 2 g/dl or**  **Need for ≥ 2 RBC** | **Ocular/**  **pericardial/**  **intraspinal/**  **intrarticular** | **Retro peritoneal** | **Muscular*** | **Requiring surgery** |
| Site of cancer |  |  |  |  |  |  |  |  |  |
| Lung, n (%) | 165 (19.8) | 2/165 (1.2) | 0/165 (0.0) | 0/165 (0.0) | 2/165 (1.2) | 0/165 (0.0) | 0/165 (0.0) | 0/165 (0.0) | 0/165 (0.0) |
| Gastrointestinal, n (%) | 175 (21.0) | 12/175 (6.9) | 1/175 (0.6) | 0/175 (0.0) | 11/175 (6.3) | 0/175 (0.0) | 0/175 (0.0) | 0/175 (0.0) | 1/175 (0.6) |
| Urogenital, n (%) | 191 (23.0) | 10/191 (5.2) | 0/191 (0.0) | 0/191 (0.0) | 9/191 (4.7) | 0/191 (0.0) | 1/191 (0.5) | 1/191 (0.5) | 3/191 (1.6) |
| Hematological, n (%) | 106 (12.7) | 9/106 (8.5) | 3/106 (2.8) | 1/106 (0.9) | 8/106 (7.6) | 2/106 (1.9) | 2/106 (1.9) | 0/106 (0.0) | 0/106 (0.0) |
| Brain, n (%) | 35 (4.2) | 3/35 (8.6) | 1/35 (2.9) | 0/35 (0.0) | 3/35 (8.6) | 0/35 (0.0) | 0/35 (0.0) | 0/35 (0.0) | 0/35 (0.0) |
| Other, n (%) | 142 (17.1) | 4/142 (2.8) | 0/142 (0.0) | 0/142 (0.0) | 4/142 (2.8) | 0/142 (0.0) | 0/142 (0.0) | 0/142 (0.0) | 1/142 (0.7) |
| Not known, n (%) | 18 (2.2) | 0/18 (0.0) | 0/18 (0.0) | 0/18 (0.0) | 0/18 (0.0) | 0/18 (0.0) | 0/18 (0.0) | 0/18 (0.0) | 0/18 (0.0) |
| Metastatic (728 pts)^a^, n (%) | 419 (57.6) | 17/419 (4.1) | 2/419 (0.5) | 1/419 (0.2) | 15/419 (3.6) | 0/419 (0.0) | 1/419 (0.2) | 1/419 (0.2) | 1/419 (0.2) |
| Ongoing CHT (791 pts)^a^, n (%) | 364 (46.0) | 12/364 (3.3) | 3/364 (0.8) | 1/364 (0.3) | 11/364 (3.0) | 1/364 (0.3) | 1/364 (0.3) | 0/364 (0.0) | 0/364 (0.0) |
| Ongoing RT (788 pts)^a^, n (%) | 128 (16.2) | 6/128 (4.7) | 2/128 (1.6) | 1/128 (0.8) | 5/128 (3.9) | 0/128 (0.0) | 0/128 (0.0) | 0/128 (0.0) | 0/128 (0.0) |

^a^Percentages were evaluated on patients with data available reported in brackets for each variable

°according to local adjudication; * with compartment syndrome
